# Serum levels of endothelial glycocalyx constituents in women at 20 weeks' gestation who later develop gestational diabetes mellitus compared to matched controls: a pilot study

**DOI:** 10.1136/bmjopen-2016-011244

**Published:** 2016-12-15

**Authors:** David S Long, Weilin Hou, Rennae S Taylor, Lesley M E McCowan

**Affiliations:** 1 Auckland Bioengineering Institute, University of Auckland, Auckland, New Zealand; 2 Department of Engineering Science, University of Auckland, Auckland, New Zealand; 3 Department of Molecular Medicine and Pathology, University of Auckland, Auckland, New Zealand; 4 Department of Obstetrics and Gynaecology, University of Auckland, Auckland, New Zealand

**Keywords:** gestational diabetes mellitus, glycocalyx, glycosaminoglycans, endothelium

## Abstract

**Objectives:**

The aim of this pilot study was to determine the serum concentration of heparan sulfate, hyaluronan, chondroitin sulfate and syndecan-1 and if these serum concentrations can be used to identify women at 20 weeks' gestation who later develop gestational diabetes mellitus (GDM).

**Design:**

Nested case–control study from Auckland, New Zealand participants in the prospective cohort Screening for Pregnancy Endpoints study.

**Setting:**

Auckland, New Zealand.

**Participants:**

20 pregnant women (70% European, 15% Indian, 10% Asian, 5% Pacific Islander) at 20 weeks' gestation without any hypertensive complications who developed GDM by existing New Zealand criteria defined as a fasting glucose ≥5.5 mmol/L and/or 2 hours ≥9.0 mmol/L after a 75 g Oral Glucose Tolerance Test. Women not meeting these criteria were excluded from this study. The patients with GDM were matched with 20 women who had uncomplicated pregnancies and negative screening for GDM and matched for ethnicity, maternal age and BMI.

**Primary and secondary outcome measures:**

The primary measures were the serum concentrations of syndecan-1, heparan sulfate, hyaluronan and chondroitin sulfate determined by quantitative ELISA. There were no secondary outcome measures.

**Results:**

Binary logistic regression was performed to determine if serum concentrations of endothelial glycocalyx layer constituents in women at 20 weeks' gestation would be useful in predicting the subsequent diagnosis of GDM. The model was not statistically significant χ^2^=12.5, df=8, p=0.13, which indicates that the model was unable to distinguish between pregnant women at 20 weeks' gestation who later developed GDM and those who did not.

**Conclusions:**

Serum concentrations of syndecan-1, heparan sulfate, hyaluronan and chondroitin sulfate in pregnant women at 20 weeks' gestation were not associated with later development of GDM. To further explore whether there is any relationship between endothelial glycocalyx constituents and GDM, the next step is to evaluate serum concentrations at the time diagnosis of GDM.

**Strengths and limitations of this study**

- This study used a nested case control study from Auckland, New Zealand participants in the prospective cohort Screening for Pregnancy Endpoints study.
- To the best of our knowledge, this is the first study to investigate serum levels of endothelial glycocalyx layer constituents in women at 20 weeks’ gestation who later developed gestational diabetes mellitus compared to matched controls.
- Two limitations of this pilot study were the study population was predominantly of European descent (70%) and the small sample size (n=20).

## Introduction

More than 50% of women of reproductive age in New Zealand (NZ) are overweight or obese when they become pregnant^1^ and gestational diabetes mellitus (GDM) is now diagnosed in ∼18% of obese pregnant women using current NZ diagnostic criteria.^2^ Since there is a continuous relationship between increasing blood glucose on the Oral Glucose Tolerance Test (OGTT) and adverse maternal and infant outcomes,^3^ lower thresholds for international diagnostic criteria have been recommended to diagnose GDM.^4^ If adopted, these new criteria would identify up to 30% of obese women as having GDM.^5^ A simple blood test that enabled early and reliable diagnosis of GDM would improve antenatal care for women by replacing a complicated diagnostic test.

Women with GDM have increased rates of pregnancy morbidity such as preeclampsia and caesarean section as well as a 50% lifetime risk of developing type-2 diabetes.^6^ GDM exposes the unborn baby to an abnormal metabolic environment with excessive nutrients, and consequently more infants are born excessively large with increased rates of birth trauma.^7^ Of great concern, GDM in pregnancy creates a vicious intergenerational cycle, which is further compounded when the mother is also obese. The resultant large infants are more likely to become obese children and adults who later develop type-2 diabetes with resultant lifelong increased healthcare costs.^8^ ^9^ This cycle further promotes health inequalities in the next generation.^3^ Earlier diagnosis of GDM, before the usual screen at 24–28 weeks, might enable earlier intervention, such as with lifestyle advice and, if required, glucose-lowering agents, with the potential to reduce the adverse health outcomes for mother and child.^10^ ^11^

A potential early marker of DM is endothelial dysfunction (impaired endothelium):^12^ ^13^ the endothelium loses the ability to maintain homoeostasis and, thus vessel health is compromised. Fundamental to protecting vessel health is the interface between circulating blood and the endothelium. Strategically located at this interface is the endothelial glycocalyx layer (EGL).^14^ The EGL is a membranous gel-like layer of proteoglycans (eg, syndecans, glypicans, perlecan and versican), glycosaminoglycans (primarily hyaluronan (HA), heparan sulfate (HS), chondroitin sulfate (CS) and dermatan sulfate), glycoproteins and plasma proteins.^14^ ^15^ Although the existence of the EGL has been known for around 70 years,^16^ for much of this time it was thought to be only a few nanometres thick and of little functional importance.^17^ However, this view has dramatically changed in recent years: (1) the full in vivo thickness of the EGL can even exceed that of the endothelium;^18^ and (2) the thickness and composition change as a function of the health of the cell—known as shedding.^12^ ^19^ ^20^ Thus, the thickness and composition of the EGL change as a function of cell health. Constituents of the EGL are shed into the circulation and the concentrations of these constituents in the circulation can be used as indicators for EGL and endothelium health.^21^

For example, Hofmann-Kiefer *et al*^22^ measured serum levels of syndecan-1, heparan sulfate and hyaluronan throughout pregnancy in women with HELLP (haemolysis, elevated liver enzymes and low platelets), as well as in healthy non-pregnant controls. Results showed increased serum concentrations of syndecan-1, heparan sulfate and hyaluronan in patients with HELLP syndrome compared to normal pregnancy at similar gestations.^22^ Lopez-Quintero *et al*^23^ showed that cultured endothelial cells exposed to hyperglycaemia decreased heparan sulfate content in the EGL. Nieuwdrop *et al*^24^ demonstrated by sublingual imaging of the microvascular glycocalyx and intravascular distribution volume of the glycocalyx that patients with type-1 diabetes have reduced EGL volume. In addition, plasma hyaluronan and hyaluranidase (an enzyme that degrades hyaluronan and indicates the capacity for EGL degradation) concentration have been shown to be higher in patients with type-2 diabetes mellitus^25^ and type-1 diabetes^24^ ^26^ ^27^ compared to healthy controls. Also, Wang *et al*^28^ showed that patients with diabetes had higher serum concentration of Syndecan-1 compared to healthy controls. These studies imply an alteration in EGL constituents of patients with diabetes.

This pilot study aims to extend previous research on EGL constituents as biomarkers for disease status by investigating whether serum concentrations of endothelial glycocalyx constituents, previously shown to shed during diabetes, can be used to identify women at 20 weeks' gestation who later develop GDM. The primary aim is to compare serum concentration levels of EGL constituents (syndecan-1 (S1), HS, HA and CS) between women who develop GDM and matched without GDM women with normal pregnancies.

### Study design

Nested case–control study from Auckland participants in the prospective cohort Screening for Pregnancy Endpoints (SCOPE) study.^29^

### Study participants, definition of GDM and matching criteria

We identified 20 participants without any hypertensive complications from the SCOPE study^29^ (http://www.scopestudy.net/) in Auckland, New Zealand who developed GDM by existing New Zealand criteria defined as a fasting glucose ≥5.5 mmol/L and/or 2 hours ≥9.0 mmol/L after a 75 g OGTT.^30^

The patients with GDM were matched with participants who had uncomplicated pregnancies and negative screening for GDM (using the definition of GDM above) and matched for (1) ethnicity (2) maternal age (age±5 years) and (c) body mass index (BMI; matched to ±3 kg/m^2^).

### Sample size and power

No previous data existed on the differences in serum concentration of EGL constituents for pregnant women with and without GDM. However, since this was a pilot, we used all GDM cases available in the SCOPE study in Auckland, New Zealand.

### Serum sample collection

Venepuncture was performed at 20±1 weeks' gestation in non-fasting participants. Serum samples were collected into BD plain serum vacutainer tubes, placed on ice and centrifuged at 2400 g at 4°C according to a standardised protocol. Serum was stored in 250 μL aliquots at −80°C within 4 hours of collection.

### Experimental methods

To assess shedding of the EGL in the circulation in women with GDM and without GDM, we quantified the concentration of the main components of the EGL:^15^ S1, HS, HA and CS by quantitative ELISA measurements. For each EGL constituent, its concentration was determined using commercially available ELISA kits, as per the manufacturer's instructions (Syndecan-1, 950.640.096, Diaclone, Besancon Cedex, France; Heparan Sulfate, CSB-E09585h, CusaBio Biotech, Hubei Province, P.R. China; Hyaluronan, DHYAL0, R&D Systems, Minneapolis, Minnesota, USA; Chondroitin Sulfate, CSB-E09587h, CusaBio Biotech, Hubei Province, P.R. China). For each target, all samples were run in triplicate, while standards were run in duplicate; samples were randomly assigned to a triplicate block on the ELISA plate. GDM cases and their matched controls were run on the same ELISA plate. All laboratory staff performing the ELISA were blinded to GDM status and participant matches.

Before the ELISA measurements were made for the cases with GDM and matched without GDM, serial dilution experiments were performed to determine an appropriate dilution factor for each target. Since the assay range for each kit was different, the corresponding serum dilutions for each EGL constituent was also different: HS—1:1, 1:2, 1:4, 1:8, 1:16, 1:32, 1:64, 1:128; HA—1:2, 1:4, 1:8, 1:16, 1:32, 1:64, 1:128; 1:256; CS: 1:100, 1:200, 1:300; 1:400, 1:500, 1:600; and S1–2:5, 1:5, 1:10, 1:20, 1:40, 1:80, 1:160, 1:320. Each sample was run in duplicate and average concentration for each dilution was calculated along with the SD. Serum collected at 20 weeks of gestation from four European women who had a negative GDM screen and an uncomplicated pregnancy was used.

### Statistical analysis

Statistical analysis was performed using IBM SPSS Statistics (V.22)*.* Mean, SD of the mean, median and IQR were calculated for each EGL constituent measured. Since most data were not normally distributed, data are presented as the median (25th centile, 75th centile). For the analysis, logistic regression was used with GDM/without GDM as the binary outcome variable; the explanatory variables were BMI, maternal age and serum concentrations of S1, HS, HA and CS. For each explanatory variable, we obtained an OR for GDM. In addition to comparing serum concentration between patients with GDM/without GDM, the serum concentration data were analysed by t*-*tests for normally distributed data; non-normal data were log-transformed and t-tests were performed. A p value <0.05 was defined as statistically significant. All staff analysing the data were blinded to GDM status.

## Results

### Study population

The study population consisted of 20 pregnant women at 20 weeks of gestation who later developed GDM and 20 controls with a negative screen for GDM and with uncomplicated pregnancies (table 1). Seventy per cent identified themselves as being of European ethnicity.

**Table 1:** Maternal age, ethnicity and BMI for the cases with GDM and matched without GDM

	Cases with GDM (n=20)			Matched cases without GDM (n=20)		
	Maternal age (years)	Ethnicity	BMI (kg/m^2^)	Maternal age (years)	Ethnicity	BMI (kg/m^2^)
1	34	European	24.5	38	European	26.4
2	27	Indian	23.8	26	Indian	21.4
3	28	European	23.7	27	European	21.9
4	33	European	32.4	28	European	32.3
5	26	Pacific Islander	34.3	21	Pacific Islander	32.2
6	35	European	30.4	35	European	30.4
7	40	European	22.4	40	European	24.5
8	35	European	28.5	39	European	26
9	38	European	31.4	40	European	29
10	29	European	22.8	31	European	21.3
11	25	European	19.9	27	European	22
12	26	Indian	26	26	Indian	23.6
13	27	European	23.8	32	European	23
14	19	European	37.3	24	European	36.7
15	33	European	24.4	30	European	23.5
16	32	European	27.2	32	European	28.7
17	29	Asian	20.4	29	Asian	19.7
18	28	Indian	25.8	24	Indian	22.8
19	33	European	24.2	29	European	21.2
20	33	Asian	27.5	31	Asian	35.4

All women were at 20 weeks of gestation.

BMI, body mass index; GDM, gestational diabetes mellitus.

The cases with GDM had a mean age of 30.5 (SD 4.98) years and a mean BMI of 26.5 (SD 4.6) kg/m^2^. The cases without GDM had a mean age of 31.2 (SD 5.4) years and mean BMI of 25.6 (SD 4.4) kg/m^2^. Random glucose median (IQR) measures in the GDM and control groups were 6.0 (5.0–6.5) mmol/L and 5.3 (5.0–6.3) mmol/L (p=0.49), respectively.

### Dilution factor experiment

**Figure 1:**
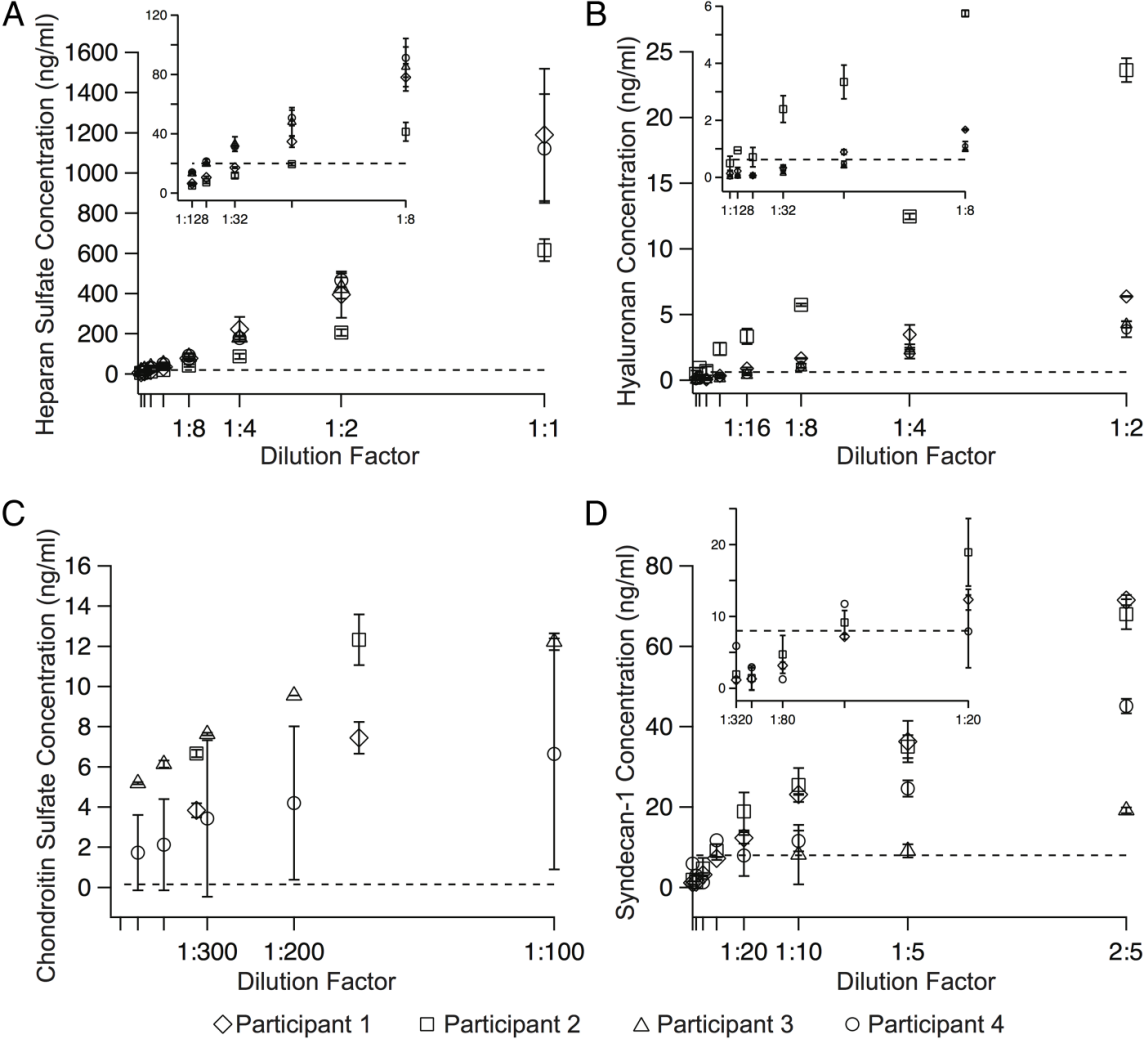
Serum concentration versus dilution factor for (A) heparan sulfate, (B) hyaluronan, (C) chondroitin sulfate and (D) syndecan-1 from the serum dilution experiments. The inset for (A), (B) and (D) shows the serum concentration versus dilution factor for the lower dilution factors used. For each dilution factor, the samples were run in duplicate and the error bars represent the standard deviation. The dashed horizontal lines represent the lower measurable range of the ELISA kit for that EGL constituent. (Note: each ELISA kit's upper measurable range is greater than the maximum value shown on the ordinate of that constituent). EGL, endothelial glycocalyx layer.

A dilution factor was recommended by the ELISA kit manufacturers for HA (1:4), CS (1:20) and S1 (1:5); however, the HS ELISA kit manufacturer did not recommend a dilution factor. To determine the appropriate HS ELISA kit dilution factor for the pilot study, and to confirm the recommended dilution factor for the other ELISA kits, a series of serial dilution experiments were performed. The serum concentration of HS (figure 1A), HA (figure 1B), CS (figure 1C) and S1 (figure 1D) was determined by ELISA in four additional SCOPE participants of European ethnicity at 20 weeks' gestation with an average maternal age of 34.5 (SD 1.7) years and average BMI of 23.3 (SD 3.5) kg/m^2^. The appropriate dilution factor range that will (1) account for individual variations in serum concentration of each constituent in the pilot study participants and (2) ensure that the serum concentrations of each constituent were within the assay's detectable range was determined to be the following: HS—1:4, 1:8, 1:16; HA—1:2, 1:4; CS—1:300, 1:400, 1:500; and S1–2:5, 1:5, 1:10. In figure 1C, only two of the four participants are shown because the serum concentration of CS at these low concentrations (<1:100) was measured for only two participants. At the higher concentrations, the serum concentration was above the detectable range (10 ng/mL) of the ELISA kit.

### Serum concentration of EGL constituents

**Figure 2:**
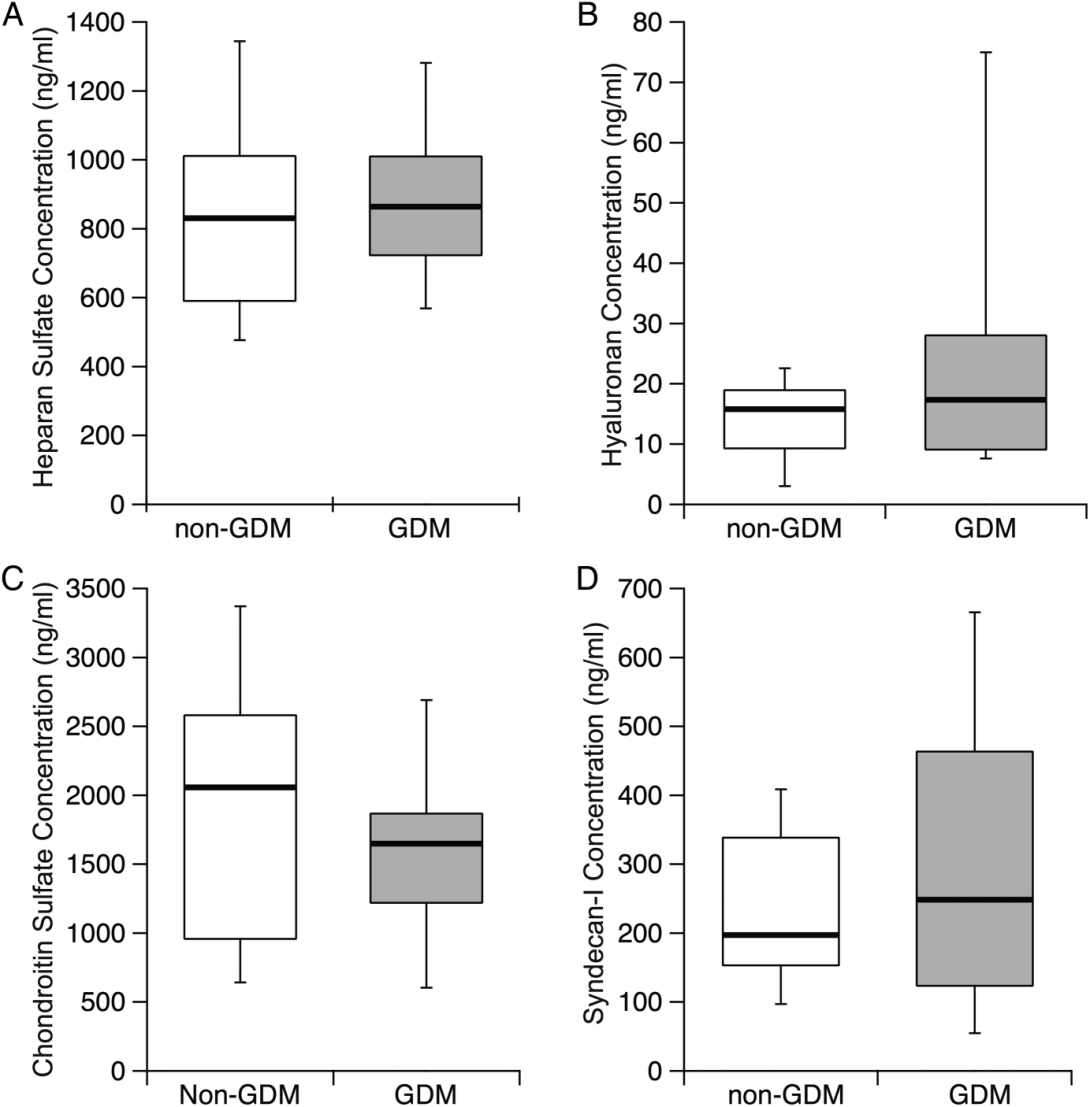
Serum concentration of (A) heparan sulfate, (B) hyaluronan, (C) chondroitin sulfate (n=10) and (D) syndecan-1 was determined by ELISA for women at 20 weeks’ gestation who either later developed GDM (grey box) or did not (white box). The black line represents the median; the top of the box represents the 75% percentile, while the bottom of the box represents the 25% percentile. No significant differences between GDM and without GDM were observed. GDM, gestational diabetes mellitus.

Serum concentration of HS, HA, CS (n=10) and S1 determined by ELISA for women at 20 weeks’ gestation who either later developed GDM, or did not, is shown in figure 2. Medians (25th centile, 75th centile) of serum concentration for each target are: (1) HS—867.7 (722.8, 1009.6) ng/mL for GDM cases versus 830.8 (590.9, 1011.4) ng/mL for matched cases without GDM; (2) HA—17.4 (9.09, 28.04) ng/mL for cases with GDM cases versus 15.81 (9.31, 18.96) ng/mL for matched without GDM; (3) CS—1648.6 (1219.8, 1866.2) ng/mL for cases with GDM versus 2056.6 (957.3, 2580.3) ng/mL for matched without GDM; and (4) syndecan-1–248.6 (123.7, 463.6) ng/mL for cases with GDM versus 197.2 (123.7, 338.4) ng/mL for matched without GDM.

No differences were observed in the log-transformed serum concentration means of HS (p=0.69, two-tailed), HA (p=0.12, two-tailed), CS (p=0.60 two tailed) and S1 (p=0.72, two-tailed) of women who later developed GDM and those who did not. After data were log-transformed, all distributions were normal; normality was assessed using the Shapiro-Wilk test (p<0.05).

Binary logistic regression was performed to determine if serum concentrations of EGL constituents in women at 20 weeks' gestation would be useful in predicting the subsequent diagnosis of GDM. The model contained five explanatory variables: maternal age, BMI and serum concentration of HS, HA and S1. Since the CS concentration was determined in only 10 of the 20 participants, it was not included in the logistic regression analysis. In addition, ethnicity was not included in the logistic regression, due to the fact that 14 of 20 participants in the pilot study identified themselves as being of the same ethnicity (European), while 3 of the participants identified themselves as Indian, 2 as Asian and 1 as a Pacific Islander. With a larger sample size, it will be possible to include ethnicity as a possible explanatory variable in the logistic regression. No potential outliers were detected. The equation met the linearity assumption for logistic regression analysis. The GDM predictive equation was p=1/(1–e^−*x*^), where x=−3.207+0.015 (maternal age in years)+0.052 (BMI in kg/m^2^)+0.047 (HA concentration in ng/mL)+0.003 (S1 concentration in ng/mL). The model was not statistically significant χ^2^=12.5, df=8, p=0.13, which indicates that the model was unable to distinguish between pregnant women at 20 weeks' gestation who later developed GDM and those who did not. The model explained between 13.8% (Cox and Snell R^2^) and 18.3% (Nagelkerke R^2^) of the variation in the development of GDM. No independent variables made a unique statistically significant contribution to the model (table 2). The cut-off value that provided the highest overall percentage of correctly classified cases was 0.5. For that cut-off value, the sensitivity and specificity (with 95% CI) were, respectively, 60% (36% to 81%) and 80% (56% to 94%).

**Table 2:** Logistic regression results (n=20), where B weights are the linear combination of the explanatory variables, SE, CI and OR is exp(B), −2LL is the negative two log likelihood, R^2^ is the proportion of variance in the outcome that the model successfully explains, χ^2^ is used to indicate how well the model fits the data, df is the degrees of freedom and p is the estimated probability of rejecting a true null hypothesis

Variable	B (SE)	p Value	95% CI for OR		
			Lower	OR	Upper
Constant	−3.207 (3.281)	0.328	–	0.040	–
Maternal age (years)	0.015 (0.075)	0.843	0.876	1.015	1.177
BMI (kg/m^2^)	0.052 (0.095)	0.584	0.875	1.053	1.268
Heparan sulfate (ng/mL)	0.000 (0.095)	0.887	0.997	1.000	1.003
Hyaluronan (ng/mL)	0.047 (0.036)	0.184	0.978	1.049	1.125
Syndecan-1 (ng/mL)	0.003 (0.002)	0.237	0.998	1.003	1.007
−2LL	49.530*				
R^2^	0.183 (Nagelkerke)	0.138 (Cox and Snell)			

χ^2^=12.499, df=8, p=0.130.

*Estimation terminated at iteration number 6 because parameter estimate changed <0.001. Initial −2LL=55 452.

df, degrees of freedom.

## Discussion

This is the first study to report HS, HA, S1 and CS serum concentration data in pregnant women at 20 weeks' gestation who either later developed GDM, compared with a control group matched by BMI and age who did not develop GDM. This pilot study showed that serum concentrations of HS, HA and S1 alone, and in combination with maternal age and BMI, were not associated with the later development of GDM.

Serum concentrations of HS, HA, S1 and CS were used for two reasons. First, these are the most prominent components of the EGL.^14^ ^21^ ^31^ ^32^ Second, the selection was based on previous studies of glycocalyx shedding in clinical settings.^33^ For instance, Nieuwdorp *et al*^24^ showed plasma levels of HA to be significantly (p<0.01) increased in male patients with type-1 diabetes compared with male patients without type-1 diabetes. In addition, Hofmann-Kiefer *et al*^34^ showed that pregnant women with HELLP syndrome had more pronounced shedding of EGL components (eg, S1, HS and HA). Finally, plasma concentration of S1, HS and HA has been demonstrated to increase after coronary artery bypass grafting.^35^ ^36^

The organisation and workflow for this pilot worked well and was divided among three different researchers. The first researcher (RT) organised the serum samples. The second researcher (DL) organised the sample layout on the ELISA plates and performed the statistical analysis (matches known, blind to GDM status). The third researcher (WH) performed the ELISA experiments and quantified the serum concentration (blind to matches and GDM status).

A limitation of this pilot study was the small sample size (n=20). To the best of our knowledge, these are the first data on serum concentration of these EGL constituents for women at 20 weeks' gestation who later developed GDM. Thus, the sample size could not have been calculated accurately a priori. The serum samples used in this pilot were from women at 20 weeks' gestation— 4–8 weeks before GDM is typically diagnosed with the OGTT. An increase in the sample size could possibly change the results/conclusions of this pilot study. As a result, conclusions drawn from these results should be interpreted bearing this in mind. However, we believe the next step should be to measure the serum concentration of EGL constituents at the time of diagnosis of GDM (time-of-disease samples). Our next step will be to measure the serum concentration of these EGL constituents later in pregnancy after diagnosis of GDM. These studies will help establish whether serum concentrations of EGL constituents are involved in the pathophysiology of GDM, at the time of disease, a necessary step before considering whether a larger study is justified.
